# A Rare Case of Acalculous Cholecystitis Secondary to Infectious Mononucleosis

**DOI:** 10.7759/cureus.93697

**Published:** 2025-10-02

**Authors:** Mithil Sheth, Thomas Barrineau

**Affiliations:** 1 Emergency Medicine, William Carey University College of Osteopathic Medicine, Hattiesburg, USA; 2 Emergency Medicine, St. Tammany Parish Hospital, Covington, USA

**Keywords:** abdominal pain, acalculous cholecystitis, cytomegalovirus, epstein-barr virus, mononucleosis, pharyngitis

## Abstract

Infectious mononucleosis (IM) is a syndrome characterized by malaise, headache, lymphadenopathy, pharyngitis, rash, enlarged spleen, and fever. Epstein-Barr virus (EBV) is the most common cause of IM, though other organisms, such as cytomegalovirus (CMV), can cause it as well. Complications include transient hepatitis, splenic rupture, peritonsillar abscess, and other organ damage.

This case study illustrates a case of acalculous cholecystitis caused by IM - a rare complication - with 44 EBV-associated acute acalculous cholecystitis (AAC) cases reported, and far fewer cases of CMV-associated AAC, though the exact number varies in the literature.

The patient was a 23-year-old woman who presented to the Emergency Department (ED) due to acute-onset right upper quadrant (RUQ) abdominal pain with radiation to her right upper back, accompanied by nausea, sore throat, and fevers. Laboratory workup and abdominal ultrasound (US) suggested AAC secondary to IM, and the patient was treated supportively.

Though AAC is an uncommon complication overall, similar cases have been reported in the literature that show overlapping clinical features; however, variability exists in laboratory findings, imaging, and associated symptoms. Presentations can differ depending on the patient’s age, immune response, and the specific viral etiology, as will be described later in the discussion.

This case highlights the importance of considering mononucleosis in the differential diagnosis of AAC, particularly in young adults presenting with symptoms consistent with a viral prodrome. Awareness of this rare complication can guide appropriate management, help avoid unnecessary interventions such as surgery, and prevent unneeded radiation and antibiotic exposure.

## Introduction

Infectious mononucleosis (IM) is a clinical syndrome most commonly caused by Epstein-Barr virus (EBV) and cytomegalovirus (CMV). Infection typically presents with low-grade fever, malaise, pharyngitis, lymphadenopathy, and splenomegaly, especially in adolescents and young adults. While the majority of cases resolve on their own, mononucleosis has been associated with complications such as splenic rupture, hematologic abnormalities, neurologic manifestations, and cancers such as Burkitt’s lymphoma [1].

This case illustrates a rare complication: acute acalculous cholecystitis (AAC) - inflammation of the gallbladder in the absence of gallstones. This occurs due to several pathophysiological mechanisms, including direct infection of endothelial and epithelial cells of the gallbladder (CMV), or infection/infiltration of B-lymphocytes (EBV), with resultant lymphoid hyperplasia; immune-mediated inflammation obstructing bile flow and leading to biliary stasis; and compromise of vasculature due to cytokines.

AAC is more commonly seen in critically ill or postoperative patients, making its occurrence in previously healthy individuals rare and diagnostically challenging, with 44 reported cases of EBV-associated AAC in the literature [2].

This case highlights a rare presentation of mononucleosis-associated AAC in a young woman who presented with right upper quadrant (RUQ) abdominal pain, pharyngitis, nausea, and systemic symptoms. 

## Case presentation

A 23-year-old female with no significant past medical history presented to the Emergency Department (ED) with acute-onset RUQ and epigastric abdominal pain radiating to the right shoulder, with associated nausea. The pain began a few hours prior to presentation and was exacerbated by food intake. She also reported a six-day history of malaise and throat swelling, as well as a four-day history of sore throat and subjective fevers. Two days prior to presentation, she had been evaluated at an urgent care clinic and prescribed amoxicillin-clavulanate for presumed streptococcal pharyngitis. Her symptoms had initially improved following treatment but worsened with the new onset of abdominal pain and nausea, prompting further evaluation.

Vital signs were normal, with a blood pressure of 122/81 mmHg, pulse of 87 beats per minute (bpm), respirations at 18 breaths per minute, temperature of 98.3°F, and oxygen saturation at 97%. The patient was in no acute distress. Throat examination revealed bilateral patchy tonsillar exudates, and there was right-sided posterior cervical lymphadenopathy. Abdominal examination demonstrated tenderness in the RUQ without rebound or guarding. Cardiac examination depicted a normal rate and rhythm, with no murmurs, rubs, or gallops. Pulmonary examination depicted clear breath sounds bilaterally. Musculoskeletal assessment showed a full range of motion in the right shoulder, with no tenderness or deformity. Cervical spine examination was normal, with a full range of motion and no tenderness. Skin examination revealed no rashes, petechiae, or bruising.

**Figure 1:**
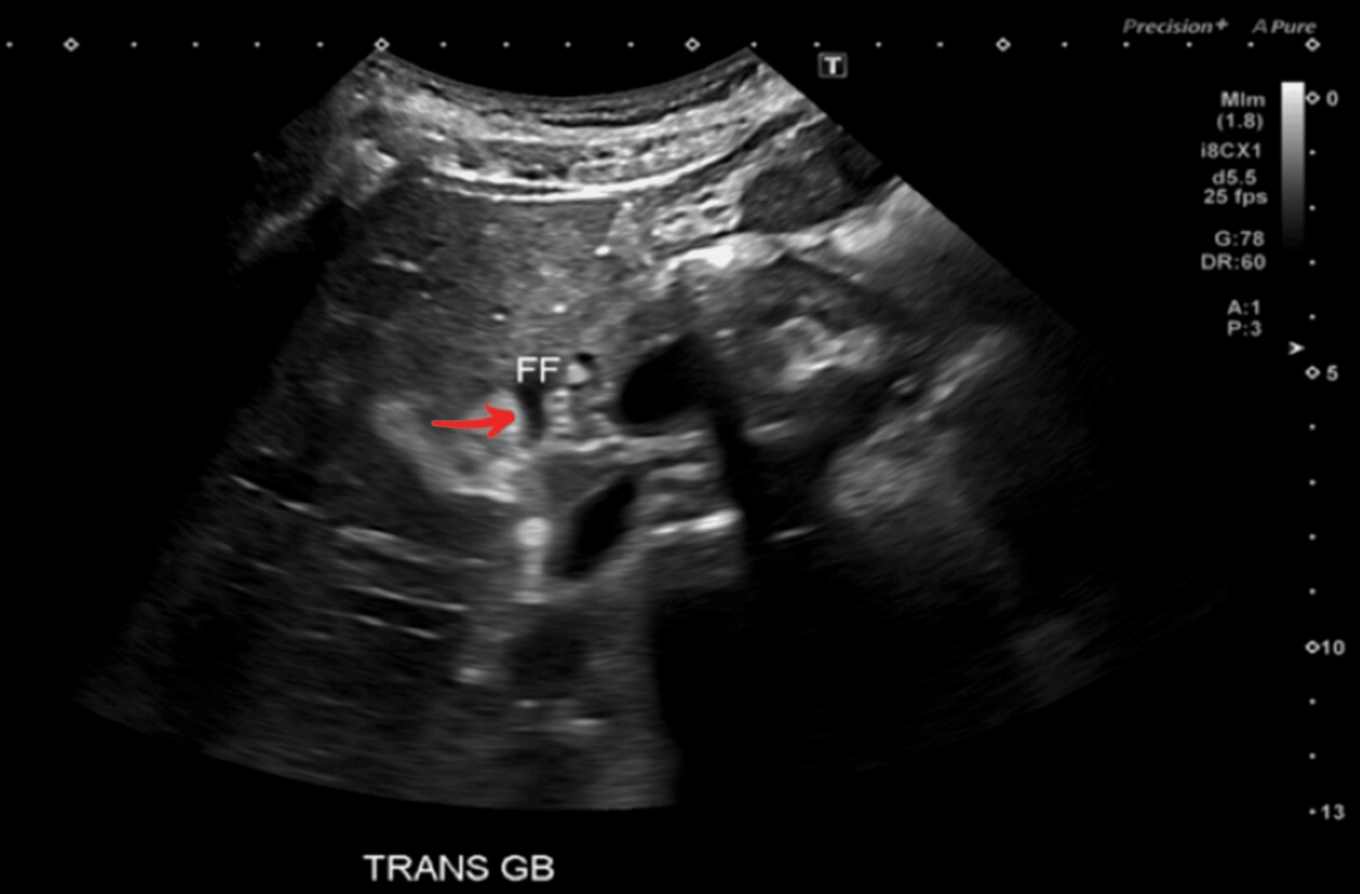
Abdominal ultrasound depicting free fluid around the gallbladder Red arrow pointing to free fluid around the gallbladder (anechoic appearance).

**Figure 2:**
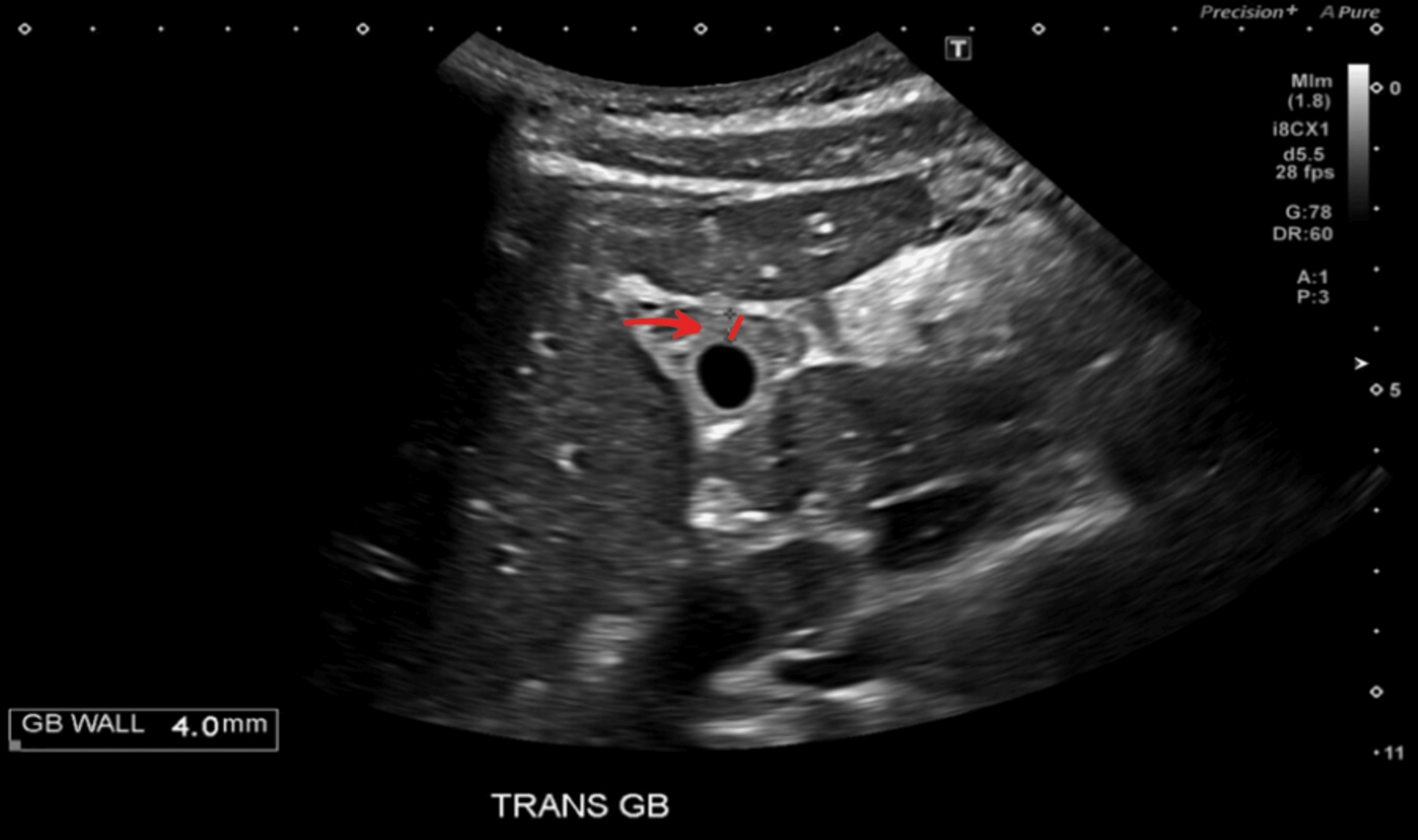
Abdominal ultrasound demonstrating increased gallbladder wall thickness at 4.0 mm Red arrow pointing to the thickened gallbladder wall (solid red line).

Abdominal ultrasound (US) demonstrated a positive sonographic Murphy’s sign, pericholecystic fluid around the gallbladder, as depicted by the red arrow in Figure 1, and gallbladder wall thickening measuring 4.0 mm, as depicted by the solid red line in Figure 2. No gallstones were identified. All in all, these imaging findings raised a strong suspicion of AAC. Abdominal computed tomography (CT) imaging was deferred, given the absence of clinical or diagnostic indications warranting further evaluation. Laboratory studies are summarized in Table 1.

**Table 1:** Lab results

Test	Result	Reference Range
Platelet Count	69,000/µL	150,000/µL-450,000/µL
White Blood Cell Count (WBC)	2.52 x 10^3^/µL	4.5 x 10^3^/µL - 11.0 x10^3^/µL
Absolute Neutrophil Count (ANC)	1.0 x 10^3^/µL	1.5 x 10^3^/µL - 8.0 x 10^3^/µL
Aminotransferase (AST)	281 U/L	10 U/L - 40 U/L
Alanine Aminotransferase (ALT)	318 U/L	10 U/L - 40 U/L
Alkaline Phosphatase (ALP)	362 U/L	30 U/L - 120 U/L

The thrombocytopenia and leukopenia seen in Table 1 further supported the diagnosis of virus-induced hepatobiliary inflammation. Serologic testing for viral hepatitis A, B, and C, as well as influenza, was negative. Beta-hCG testing was negative. A Monospot test was positive. Peripheral blood smear demonstrated increased reactive lymphocytes.

In the ED, the patient received intravenous ketorolac (15 mg), morphine (4 mg), and ondansetron (4 mg) for symptom management. Antibiotics were not given due to the viral etiology of the disease, given the thrombocytopenia, leukopenia, and positive Monospot testing. Surgical consultation was not pursued, as the clinical presentation and diagnostic findings did not suggest a need for operative intervention. She was admitted for observation and supportive care. A low-fat diet was initiated, and she was managed with continued antiemetics and analgesics. By hospital day 2, the patient demonstrated significant improvement in her laboratory findings, including improved white blood cell (WBC) count, absolute neutrophil count (ANC), and liver function tests (LFTs). Interestingly, her platelet count dropped from 69,000/µL to 56,000/µL, but rose to the normal range by discharge. She was discharged on hospital day 2 with a prescription for oral ketorolac, 10 mg as needed for pain. Discharge instructions included avoiding contact sports for three weeks, abstaining from alcohol, limiting acetaminophen to less than 2 grams per day, avoiding raw seafood, and continuing a low-fat diet. She was advised to follow up with her primary care provider (PCP) within two weeks for repeat laboratory testing.

Two weeks post-discharge, the patient’s serologic results for CMV and EBV are seen in Table 2.

**Table 2:** Serologic testing results for CMV and EBV CMV, cytomegalovirus; EBV, Epstein-Barr virus

Laboratory Test	Patient’s Value	Reference Range
CMV IgM antibodies	1.710	<0.70
CMV IgG antibodies	473.0	<0.80
EBV viral capsid antigen IgG	40.9 U/mL	<18.0 U/mL
EBV viral capsid antigen IgM	82.1 U/mL	<36.0 U/mL

Despite these findings, the patient reported complete resolution of symptoms and did not require antiviral treatment. Her other laboratory studies showed marked improvement in WBC count, platelet count, ANC, LFTs, and alkaline phosphatase (ALP).

At her two-week follow-up, the patient reported significant improvement in her symptoms following supportive care and expressed relief that surgical intervention was not necessary. She was also grateful that she did not receive CT exposure or unnecessary antibiotic treatment. The patient provided written consent to participate in this case report.

## Discussion

IM is a syndrome characterized by malaise, headache, lymphadenopathy, pharyngitis, rash, enlarged spleen, and fever. Two important causative pathogens are EBV and CMV, with EBV most commonly implicated. EBV is a herpesvirus that primarily affects B-lymphocytes and epithelial cells, and is classically spread through salivary secretions. Infection with EBV is extremely common worldwide, with close to 95% of adults infected at some point in their lives [3]. CMV is also a herpesvirus and presents with symptoms similar to EBV. Systemic complications, including hepatitis, cytopenias, and neurologic syndromes, are possible. AAC is a lesser-known complication of this infection. It involves inflammation of the gallbladder without the presence of gallstones, and is commonly seen in critically ill (such as those with shock or severe burns) or postoperative patients. The pathophysiology of AAC includes bile stasis and/or ischemia. EBV infects B-lymphocytes and epithelial cells, triggering an inflammatory response that leads to lymphoid tissue hyperplasia, vessel injury, bile stasis, and infiltration of the gallbladder wall by immune cells. CMV causes direct cell injury to endothelial and epithelial cells, leading to ischemia and inflammation of the gallbladder wall. These mechanisms can impair bile flow, resulting in bile stasis. Furthermore, the resultant inflammation can increase vascular permeability, causing fluid shifts and further congestion, which impair flow and worsen gallbladder function. Treatment for uncomplicated AAC typically begins with supportive care and observation for 24-48 hours, along with broad-spectrum IV antibiotics. Patients who do not improve typically require percutaneous cholecystostomy in addition to IV antibiotics. If complicated cholecystitis is present (when the integrity of the gallbladder is threatened or compromised), patients require cholecystectomy [4].

The occurrence of IM causing AAC in otherwise healthy individuals is rare, with only 44 cases of EBV-associated AAC having been reported in the literature [2], and even fewer cases of CMV-associated acalculous cholecystitis. In one study involving 171 pediatric patients, overlapping features with the current case were seen, including abdominal pain, fever, elevated liver enzymes, and gallbladder wall thickening. However, differences existed: the common bile duct was significantly dilated, and bilateral eyelid edema was present in the pediatric cases - findings not seen in the current case [5]. It is important to note, however, that adult presentations may vary from those in pediatric populations.

Three recently reported cases also share features with the current presentation. One involved a 35-year-old male who presented with headache and fever, without sore throat, nausea, or abdominal pain [6]. Imaging revealed gallbladder wall thickening, and EBV was confirmed serologically. While AAC due to EBV was diagnosed, his presentation differed significantly from that of our patient, who exhibited prominent gastrointestinal and pharyngeal symptoms. Another case described a young woman with RUQ abdominal pain, sore throat, and enlarged tonsils with exudates - symptoms also seen in our patient - though her lab work showed elevated WBC and normal neutrophil count (contrasting with the leukopenia and neutropenia in this case) [7]. Finally, a rare case of CMV-associated AAC presented with epigastric pain, fever, hepatomegaly, and atypical lymphocytosis, but notably lacked pharyngitis and lymphadenopathy, two hallmark features present in our patient [8]. Table 3 compares and contrasts these findings in the literature with the findings of the current case. Together, these comparisons to established recent cases underscore the importance of recognizing both shared and variable features in AAC secondary to viral infections, especially EBV and CMV. 

**Table 3:** Similarities and differences between the current case and previously reported cases of EBV and CMV-associated AAC GI, gastrointestinal; EBV, Epstein-Barr virus; CMV, cytomegalovirus; RUQ, right upper quadrant; LFTs, liver function tests; WBC, white blood cell count; AAC, acute acalculous cholecystitis

Category	Current Case	Pediatric Study (n = 171)	Case 1: 35-Year-Old-Male (EBV)	Case 2: Young Woman (EBV)	Case 3: CMV Case
Symptoms	Abdominal pain, sore throat, fever, pharyngitis, lymphadenopathy, tonsillar exudates	Abdominal pain, fever	Headache, fever (no GI or pharyngeal symptoms)	RUQ pain, sore throat, tonsillar exudates	Epigastric pain, fever (no pharyngitis/lymphadenopathy)
Imaging	Gallbladder wall thickening; no common bile duct dilation	Gallbladder wall thickening; common bile duct dilation	Gallbladder wall thickening with no gallstones	Gallbladder wall edema with no gallstones	Gallbladder wall thickening, no gallstones
Serologies/laboratory studies	EBV/CMV suspected; leukopenia, neutropenia, elevated LFTs	Elevated LFTs	EBV+ serology	EBV+; elevated WBC, normal neutrophil count	CMV+; atypical lymphocytosis, hepatomegaly
Distinguishing features	Prominent pharyngeal symptoms; hematologic abnormalities	Pediatric age, common bile duct dilation and bilateral eyelid edema	No GI or pharyngeal symptoms	Similar symptoms; no neutropenia or leukopenia	Lacks pharyngeal signs; CMV-specific

## Conclusions

In the current case, both EBV and CMV testing returned positive, making it especially unique, as most cases identify either EBV or CMV as the causative agent. However, based on the serologic findings, recent or acute EBV infection is most likely - especially due to the markedly elevated EBV viral capsid antigen IgM. The elevated CMV results most likely represent false-positive results, which are commonly seen in acute EBV infection. Cross-reactivity is the most likely cause of the elevated CMV serologic results and not an indicator of acute infection, especially given the high IgG CMV antibodies. However, given the overlap in clinical presentation and the positive results for both viruses, it is important to consider that either - or both - of these agents may have contributed to the disease process. Nonetheless, clinicians should remain aware of viral causes of cholecystitis, particularly in young, otherwise healthy individuals presenting with atypical features and systemic symptoms.

This case underscores the importance of maintaining a broad differential diagnosis in patients presenting with abdominal pain, especially when accompanied by symptoms such as pharyngitis and lymphadenopathy. Clinicians should be aware of mononucleosis as a potential cause of AAC and should pursue confirmatory testing before initiating unnecessary interventions. Recognition of this rare complication allows for effective management with a conservative approach - such as with this patient - and prevents avoidable morbidity associated with misdiagnosis or overtreatment.
